# When theory beats practice: the implementation of competency-based education at healthcare workplaces: Focus group interviews with students, mentors, and educators of six healthcare disciplines

**DOI:** 10.1186/s12909-023-04446-3

**Published:** 2023-06-29

**Authors:** Oona Janssens, Mieke Embo, Martin Valcke, Leen Haerens

**Affiliations:** 1 Department of Educational Studies, Faculty of Psychology and Educational Sciences, Ghent University, H. Dunantlaan 2, Ghent, 9000 Belgium; 2 Department of Movement and Sports Sciences, Faculty of Medicine and Health Sciences, Ghent University, Ghent, 9000 Belgium; 3 Expertise Network Health and Care, Artevelde University of Applied Sciences, Voetweg 66, Ghent, 9000 Belgium

**Keywords:** Competency-based education, Healthcare education, Multidisciplinary research, Work-integrated learning

## Abstract

**Background:**

Work-integrated learning constitutes a large part of current healthcare education. During the last decades, a competency-based educational (CBE) approach has been introduced to reduce the theory-practice gap and to promote continuous competency development. Different frameworks and models have been developed to support CBE implementation in practice. Although CBE is now well-established, implementation at healthcare workplaces remains complex and controversial. This study aims to explore how students, mentors, and educators from different healthcare disciplines perceive the implementation of CBE at the workplace. The six-step model of Embo et al. (2015) was used as a base: (1) competency selection, (2) formulating learning goals, (3) self-monitoring performance, (4) self-assessing competency development, (5) summative assessment of individual competencies, and (6) summative assessment of global professional competence.

**Methods:**

Three semi-structured focus group interviews were conducted with (1) five students, (2) five mentors, and (3) five educators. We recruited participants from six different educational programs: audiology, midwifery, nursing (associate degree and bachelor), occupational therapy, or speech therapy. We used thematic analysis combining an inductive and deductive approach.

**Results:**

An overview of the predefined competencies was hard to find which complicated CBE implementation and resulted in a lack of consistency between the steps; e.g., the link between the selection of relevant competencies (step 1) and the formulation of learning goals based on these selected competencies (step 2) was absent. Furthermore, the analysis of the data helped identifying seven barriers for CBE implementation: (1) a gap between the educational program and the workplace, (2) a lacking overview of predefined competencies, (3) a major focus on technical competencies at the expense of generic competencies, (4) weak formulation of the learning goals, (5) obstacles related to reflection, (6) low feedback quality, and (7) perceived subjectivity of the assessment approach.

**Conclusion:**

The present barriers to CBE implementation lead to a fragmentation of current work-integrated learning. In this way, theory beats practice when it comes to CBE implementation as the theory of CBE is not effectively implemented. However, the identification of these barriers might help to find solutions to optimize CBE implementation. Future research seems critical to optimize CBE so that theory can meet practice and the opportunities of CBE optimize healthcare education.

**Supplementary Information:**

The online version contains supplementary material available at 10.1186/s12909-023-04446-3.

## Background

Nowadays, work-integrated learning has become an integral part of healthcare education for many reasons. First, learners experience many positive outcomes of work-integrated learning. It better prepares them for the external demands from the healthcare system and regulatory organizations, and for responding to the societal expectations towards professionals [1]. Work-integrated learning is often approached as an umbrella term for pedagogical methods emphasizing integration of theory and practice [2, 3]. Although it is difficult to find a uniform definition of the concept, work-integrated learning has been introduced a long time ago [4]. The origin of work-integrated learning can be traced back from an empiricist epistemology that focused on actively constructing knowledge, skills, and attitudes on the base of authentic experiences [5, 6]. It is a less formal approach of education as it is mostly unplanned, highly collaborative and opportunistic, and it largely depends on the context. Nevertheless, work-integrated learning is strongly connected to formal learning at educational institutions with the competencies that are formally learned being applied and transferred to the workplace [4]. In an education setting, work-integrated learning implies that students actively construct their knowledge at the workplace [5, 6]. Although the educational value of learning in authentic contexts is widely accepted, work-integrated learning remains a complex process and several approaches have been implemented to support this process, with competency-based education (CBE) being the most known.

A competency comprises the integration of knowledge, skills, and attitudes in a specific context. When a curriculum adopts a CBE approach, this often results in the provision of tasks that require the combined activation and adoption of knowledge, skills, and attitudes in the task context [7]. In CBE, the focus changes from time-based input (hours of curriculum representation) to achieved output (predefined competencies). Students are stimulated to attain all competencies during the full educational continuum instead of a limited time window or internship [8, 9]. To effectively implement CBE, a clear definition is required of which competencies students need in order to meet societal and patient needs, and to support their learning processes [10]. The transition towards CBE requires healthcare educational programs to restructure their curriculum and learning environments [11].

To support this transition, many competency frameworks are available. Widely used examples are the Canadian Medical Education Directives for Specialists (CanMEDS) [12], the Tomorrow’s Doctors [13], the Scottish Doctor [14], and the Accreditation Council for Graduate Medical Education (ACGME) [15]. Some educational programs use these frameworks or build on a redesign of the frameworks listed above [16–24]. When implementing competency frameworks in practice, related behavioral indicators or assessment criteria are used to assess competencies and determine mastery levels. These indicators help operationalizing the competencies and help capturing learner’s progress [25, 26]. Using competency frameworks and related indicators offers the advantage to monitor student’s progress; e.g., by the integration in (e)portfolios. ePortfolios serve as online learning spaces where students can reflect on their learning journey. ePortfolios contain centralized collections of work on which students can be assessed and or through which they can showcase their accomplishments to potential employers [27]. ePortfolios can support competency-based education (CBE) as these can serve as a tool to scaffold the learning process and to support the students as well as the educator or mentor [28]. Nevertheless, this requires an adequate integration of CBE in work-integrated learning.

Next to the content of CBE that needs to be implemented, the way in which CBE is implemented, is crucial. Implementation science can be helpful in this respect as it includes “a scientific study of methods to promote the systematic uptake of research findings and other evidence-based practices into routine practice to improve the quality and effectiveness of healthcare services” [51]. Roger’s Diffusion framework Of Innovations (DOI) research examines the contextual factors influencing implementation within complex organizations. It identifies four elements: the communication channel, the social environment, the innovation itself, and the available time which contribute to successful transfer and implementation [51].

**Fig. 1:**
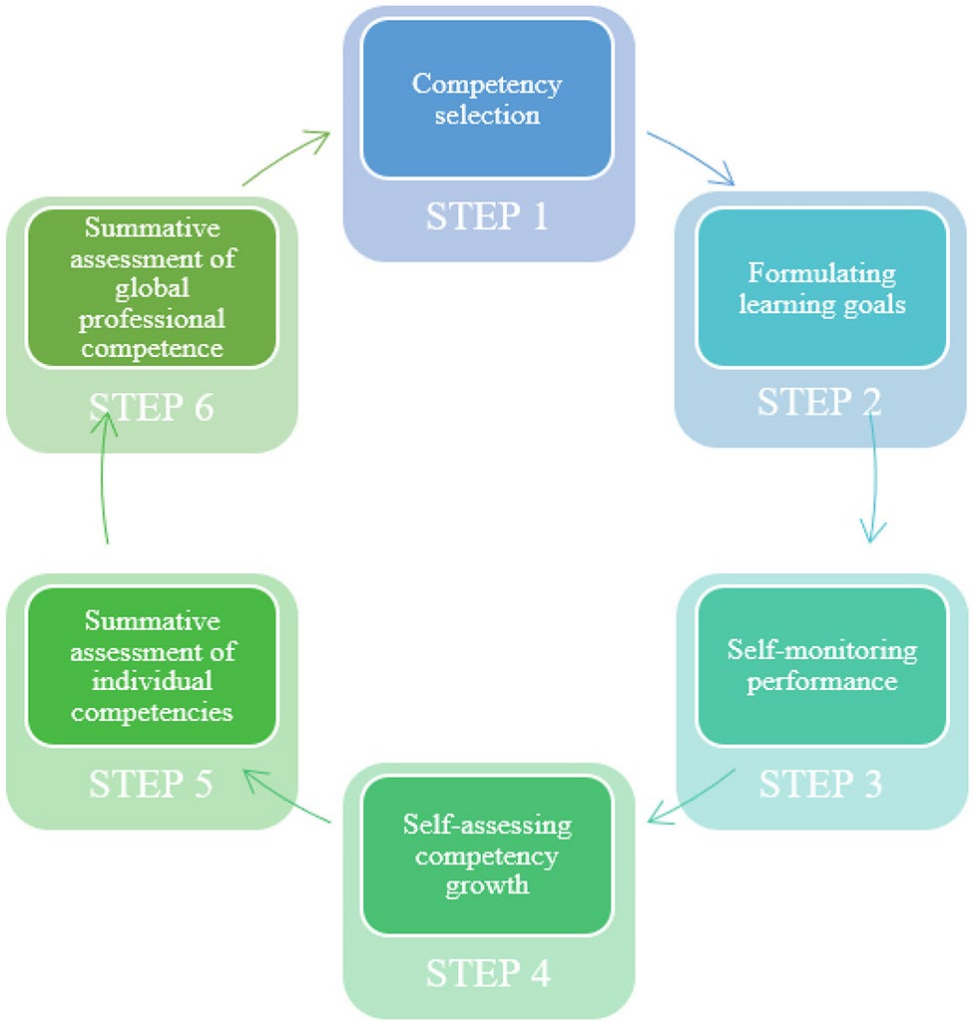
Visualization of the six-step model of Embo et al. (2015)

When it comes to the innovation itself, namely CBE, several models were created to support effective implementation. The six-step model of Embo et al. (2015) was developed in view of healthcare education to support the continuous and self-directed learning process of students at the workplace. The model consists of six steps: (1) competency selection, (2) formulating learning goals, (3) self-monitoring performance, (4) self-assessing competency development, (5) assessment of individual competencies, and (6) assessment of global professional competence (see Fig. 1) [29].

To pursue continuity throughout the educational program and to capture competency development, internal consistency between the steps is necessary. First, predefined competencies and learning goals should be aligned; then the assessment should be aligned with the learning goals, and, lastly, the feedback should be sufficiently rich to cover the full learning goal spectrum.

The steps of the model are expected to guarantee gradual attainment of the predefined competencies as stipulated in CBE. To clarify, after selecting the competencies that can effectively be trained and assessed during an internship *(step one)*, the formulation of learning goals *(step two)* might continue the work-integrated learning process [30]. This guarantees deliberate practice that is the base for reflection on these experiences [31]. The ‘self-monitoring performance’ step *(step three)* contains a reflection and feedback aspect. Students are expected to take the initiative to reflect *on daily performances* and compare these reflections with competency standards. Moreover, they are stimulated to ask for feedback on these daily performances while mentors are expected to foster reflection and provide feedback after reading and/or discussing the reflections with the student [29]. The daily reflections should improve performances during patient care and support expertise development [30, 32]. To further optimize competency development, a second reflection activity is foreseen in *step four*, self-assessing competency development. In this step, students reflect *on progress* over a longer period of time, and the educator *and* mentor provide feedback on this progress. This reflection on the development of competencies is essential for continuous competency development, albeit this process is more complex and abstract. Next to the formative part of assessment, summative assessment of individual competencies *(step five)* is indispensable in the model and mostly takes place at the end of an internship. Summative assessment of global professional competence *(step six)* is also of great importance. It is an overall judgment about competence and/or fitness for practice, and mostly happens at the end of each program year [31, 33]. However, the iterative character of the model states that summative assessments alone may not provide sufficient feedback to drive learning and that both formative and summative assessment types are needed to assure that students meet the predefined competencies and are ‘fit for practice’ at graduation [29, 31].

CBE is expected to optimize educational experiences thanks to the strong focus on outcomes related to clearly defined competencies. The CBE advantages could provide a transparent training approach for healthcare professionals to guarantee competency development [34]. However, CBE implementation is challenging when looking at the required curriculum, available learning environment adaptations, and support tools such as (e)portfolios. CBE is often weakly implemented and rather invokes a large administrative burden [34]. To explore implementation issues of CBE at the workplace, we build on the perspectives of students, mentors, and educators from different healthcare educational programs in Flanders (Belgium). Therefore, this study aims to answer the following research question:

*What are the perceptions of students, mentors, and educators from different healthcare disciplines about the implementation of CBE in current practice at the workplace?*

## Methodology

### Context

This study was conducted in February and March 2020 in Flanders (Belgium) with students, mentors, and educators from different healthcare programs as they are the key stakeholders during work-integrated learning [29]. The work-integrated learning literature is complex because of an overwhelming amount of context-specific roles and responsibilities. Therefore, we provide definitions of the stakeholders clarifying their role in the context of this study (Table 1).

**Table 1:** The definitions of stakeholders

Stakeholder	Definition in the context of this study
*Student*	A person who is being trained at the workplace to become a healthcare professional.
*Mentor*	A healthcare professional working at the workplace training students during work-integrated learning.
*Educator*	A person affiliated to a specific healthcare educational institution and guiding students to achieve their predefined competencies.

### Design

To answer the research question, we set up a descriptive qualitative study involving multidisciplinary teams by conducting semi-structured focus group interviews.

### Sample

Participants representing six different educational programs (Table 2) and three stakeholder groups (student, mentor, and educator) were invited to participate. For the stakeholder group of students, only third-year students were invited to guarantee valuable experience with work-integrated learning. Mentors and educators were included when they felt to have sufficient experience with guiding students during work-integrated learning. Therefore, a non-probability sampling method was used to select participants purposively [35]. Participants were contacted through e-mail after having received their contact information via the coordinators of included educational programs. They were invited to participate after having received information about the study. Twenty-three persons were invited through e-mail to participate of which fifteen agreed. Persons deciding not to participate experienced a high workload and were short in time. As such, non-participation was not related to the topic of the study.

The first focus group interview was conducted with five students, the second with five educators, and the third with five mentors. Stakeholder groups were not mixed up because one group could influence another and the input of all stakeholder groups was chased. An overview of the sample can be found in Table 2. There are a lot of differences between countries and even educational institutions when it comes to healthcare educational programs. To harmonize these large differences, we adopted the European Qualifications Framework (EQF) so that understanding is facilitated and country-specific program labels are reduced (https://europa.eu/europass/en/compare-qualifications). Three of the participants identified themselves as male, the others as female. The male/female ratio reflects the real-life situation in which over 75% of the healthcare professionals is reported being female [37, 36].

**Table 2:** Overview of the sample

	Students		Mentors		Educators	
	♂	♀	♂	♀	♂	♀
Nursing (EQF level five)	n = 2			n = 1		
Nursing (EQF level six)		n = 1		n = 1		n = 2
Midwifery (EQF level six)			n = 1			n = 1
Occupational therapy (EQF level six)				n = 1		n = 1
Speech therapy (EQF level six)	n = 1			n = 1		n = 1
Audiology (EQF level six)		n = 1				
**TOTAL**	n = 5		n = 5		n = 5	

### Data collection

The interviews were moderated by the main author (O.J.) and supported by a second person (A.A.). The interviews started with a video, visualizing the six-step model [29, 38]. This helped to develop a ‘shared language’ for the interview and to visualize the CBE building blocks [39]. A semi-structured topic guide was used, based on the six-step model of Embo et al. (2015), consisting of six questions referencing to the individual steps in the model: ‘Do you recognize this step in your work-integrated learning practice?’.

### Data-analysis

All interviews were tape-recorded with participants’ permission. Recordings were transcribed and coded using NVivo12©. A combination of an inductive and deductive thematic analysis was used because a data-driven analysis was chased (inductive) but the analysis was steered by the six-step model (deductive) [40, 41]. Because participants often gave input to other steps than the step that was discussed at that moment, the analysis started by open coding of individual units of analysis moving from specific to general coding (inductive). In the next phase, the individual codes were scrutinized to develop comprehensive themes. This hierarchical coding process allowed an analysis of data at different levels of specificity with higher-level themes providing an overview of lower-level themes [40]. The final codebook was passed to a second, independent researcher (H.D.). Up to 15% of the interviews was double-coded to control coding reliability as it is seen as a more efficient approach than double coding all data [42]. Both sets were compared and discussed. In case of disparities, causes for the disagreement were discussed and dissolved to get consensus [40]. Afterwards, the themes were assigned to the six steps of the model (deductive). As an example, the codebook of the focus group interviews with mentors was added in Appendix 1. Moreover, barriers to CBE implementation were detected by getting deeply immersed in the data, interpreting codes and themes, and making links between themes. In this way, patterns throughout the data were detected, and the significance of these patterns and their broader meanings and implications were explored in relation to the literature [40].

## Results

Overall, the participants strongly recognized the steps of the model of Embo et al. (2015) in their work-integrated learning practice. However, they admitted that one step did not take place in practice: self-assessing competency development (*step 4*). Each step will be outlined in the following section. Afterwards, the interconnection among the steps will be separately illustrated.

### Step 1: competency selection

The first step, the selection of relevant competencies out of the program’s competency framework, was highly valued by educators, while mentors stated that the predefined competencies were not often used in practice, albeit recognizing their importance and usefulness.

*“The competencies are there but they are not named at the start of an internship. Because they (students) don’t think the competencies are important. For both students as mentors. Although it is very important to take into account the competencies. It is that evolution we want to take into account.”* (mentor, midwifery).

For students, it was necessary to get a clear view of the predefined competencies at the start of their internship and to bring them in relationship with the competencies of the full program. Although they were encouraged by educators to plan their learning based on the predefined competencies, students mentioned that this overview was hard to find.

*“You have to do your very best to find… I don’t think that… It could be more eye-catching.”* (student, nursing).

One of the problems regarding the integration of competencies was competency conceptualization. Students and mentors used the words ‘talents’ or ‘gifts’ when they were talking about competencies. Students claimed that more attention should be paid to ‘personal’ competencies in the sense of talents and emotions. They mentioned that the focus on predefined competencies was often too strong and a more holistic vision was needed.

*“…because the educational program focuses very strongly on communicative competencies and all the rest… And, you also grow rapidly as a person within your educational program. So, I think it would be very interesting to show how you are as a person at the beginning of your education.”* (student, nursing).

### Step 2: formulating learning goals

In the beginning of internships, formulating learning goals was perceived as highly important by all participants, but also time consuming. Especially, mentors and educators stated that they lacked time to tackle this step in practice.

Another problem was that learning goals were often formulated in such a way that profound connections with daily practice were lacking.

*“Earlier, we were working with an expectation pattern like what do I expect for myself during my internship? I think this is less clear when formulating learning goals nowadays. Those learning goals are like more artificial for me. That’s what I found less interesting about it.”* (student, associate degree nursing).

Furthermore, students mentioned they missed the development angle when formulating learning goals, because they perceived that they needed to build on the fixed predefined competencies rather than formulating personal learning goals after a profound reflection on personal learning gaps.

Moreover, students often focused on technical competencies when formulating learning goals while mentors and educators focused also on generic competencies as well. The emphasis on both technical and generic competencies can be linked to the conceptualization of the T-shaped professional model [43]. Within this model, technical competencies refer to discipline-specific competencies and generic competencies refer to more general, cross-disciplinary competencies [44, 45].

*“With us, I find the formulation of learning goals of poor quality because they have to formulate two or three learning goals and then you have a lot of students saying they want to place a drip three times during that specific internship. In that way, you are missing… some students might make better learning goals.”* (educator, midwifery).

### Step 3: self-monitoring performance (= reflection on daily performances)

On the one hand, students perceived daily reflections on performances as a compulsory activity, instead of being an integrated part of the learning process. None of the participating students were intrinsically motivated to write reflections. They admitted that the reflections were necessary to get a good assessment. They also stated that there was hardly sufficient time for writing reflections during an internship.

*“And when is there time for reflection during your internship?”* (moderator).

*“That’s only at the moment of assessment now and then.”* (student, nursing).

On the other hand, mentors and educators expressed that students’ reflections were of poor quality. There was a strong focus on technical competencies over generic competencies. Mentors perceived this as a substantial problem because generic competencies were often forgotten and the ‘how’ and ‘why’ of what students did might therefore be considered secondary. Furthermore, mentors and educators noticed a lack of *deep* reflection as students often seemed to write superficially.

*“You see them often writing about techniques and actions, like ‘I have done this and this but how they did it and how they felt about it’… That is strongly lacking.”* (mentor, nursing).

*“We, as educators, receive the guideline from the educational program that when students do not reflect on a certain competency and when they do not write feedback that they will receive an unsatisfactory grade. And the students know that very well so the reflections become more and more a piece of proof instead of a real reflection.”* (educator, midwifery).

Regarding feedback, students perceived receiving feedback as one of the most important facilitators of their learning process. Nevertheless, lack of time to give feedback was perceived as a daily barrier by mentors and educators. Consequently, students felt that the limited and poor feedback was ineffective to facilitate their learning process.

*“In combination with ‘please, you can always write down some feedback’… I would like to, but time is often lacking to do that.”* (mentor, midwifery).

*“The feedback I am receiving from the educator is given pretty quickly so that could be much more comprehensive and longer. The two times I see them are too little for someone who is assessing me. I think they don’t know me and I can’t properly defend myself in several areas.”* (student, audiology).

Another problem with feedback presented by educators and mentors was that a substantial amount of feedback was focused on technical competencies rather than generic competencies. They both stated that the former feedback was easier to give.

*“That is the reason why mentors… avoid is a big word, but giving feedback on a badly executed wound care is easy like ‘like this, like that, these are the guidelines’ but giving feedback on attitude or other skills is a whole other story.”* (mentor, midwifery).

*“But I, I speak for myself, but I catch myself focusing on the more technical part than on the more communicative part while giving feedback. But I notice that for me, the focus is automatically switched to the purely technical part.”* (educator, speech therapy).

Accordingly, mentors refrained from hurting students’ feelings by giving negative feedback. They explained that the healthcare sector is a soft domain where emotions and feelings should be considered. However, neglecting negative feedback hinders the effectiveness of the feedback process because a part of the base to redirect is lacking in the feedback information.

*“…that is not who we are… to hurt, so you want to formulate everything in a sensitive, careful way so that it is not perceived as negative but in a way that allows growing.”* (mentor, nursing).

### Step 4: self-assessing competency development (= reflection on competency development)

None of the stakeholders recognized this step in practice. Reflection on daily performances (step 3) during the previous step had high priority over reflection on competency development (step 4).

Nevertheless, students and mentors stated that this developmental aspect should be extended to a larger extent. Even more, not only competency development *within* an internship but also *between* internships should be emphasized more in practice according to all stakeholders. It was additionally stated that the visualization of competency development might be of added value. Students mentioned that this visualization could also help them in their future employment because employers could more easily grasp a candidate’s competence mastery. In practice, capturing this growth seemed hard, especially when also contradictory findings were reported. On the one hand, some mentors and educators advocated adopting a longitudinal approach to build on information covering all education years to grasp an image of the competence mastery of their students and to map continuity in competency development.

*“I do think that reflections on competency development could be optimized.”* (student, associate degree nursing).

*“I think it would be a good idea for students to make a report of their competencies at the end of each internship and that this report is taken to the consecutive internships.”* (mentor, occupational therapy).

On the other hand, some educators argued that evaluative information from former internships should be restricted to protect students by avoiding the risk of creating a bias or wrong expectations. Students should be able to start with a clean slate. They suggested that this information was only supposed to play a role in case of problematic competency development. They also worried about the extra work for mentors if they should read earlier internship documentation.

*“I would rather prefer that it is closed after every internship because of… sometimes, in the beginning you think ‘oh my god, how much text do I have to read’ and then you see it’s from the previous internship…”* (educator, occupational therapy).

*“Oh, that student has a 12/20 and that one has a 16/20, then you will create expectations which you can’t meet.”* (student, audiology).

### Step 5: summative assessment of individual competencies

All participants perceived this step as indispensable in practice. They believed that it was important that all stakeholder groups were involved in the assessment process to reduce incongruence between educator and mentor. Students described how they perceived a different engagement from mentors and educators in assessment. They expected a descriptive assessment from the mentor while giving grades was considered the educator’s task.

*“We have the decisive… Well, it has all to be nicely framed and argued or otherwise… They can’t receive an ‘excellent’ from the mentor for a specific key competency and an ‘inadequate’ from me. That is not correct. So then, there has to be a negotiation…”* (educator, associate degree nursing).

Mentors stated that the competencies achieved or not mostly became already clear during the internship and that surprises at the time of assessment were rare because they closely guided their students. Nevertheless, mentors and educators believed that elements of subjectivity in assessing students could cause problems:

*“Because it is always open for interpretation and then you have differences… An ‘excellent’ for me is maybe not the same as the ‘excellent’ for someone else…”* (educator, nursing).

*“It is also difficult to judge… Is that student good enough in communication? That is often gut feeling… And that is often right but not totally correct…”* (mentor, midwifery).

### Step 6: summative assessment of global professional competence

Students and mentors believed this overarching assessment perspective was part of the grading rather than a holistic appreciation of competence mastery. Students referred to the summative assessment of individual competencies (*step 5*) when discussing the relevance of this step. They were well aware of the final judgement of educators but less of the opportunities to ask and receive overall feedback to support their further competency development within and/or between future internships.

### Links between the steps

The results showed a lack of consistency between all the steps. Students and mentors found that an overview of the predefined competencies was hard to find which complicated the essential consistency between the steps. The link between the selection of relevant competencies at the start of an internship (step 1) and the formulation of learning goals based on these selected competencies (step 2) was absent. Self-monitoring daily practice (step 3) was well-established but self-assessing competency development (step 4) was missing, causing a developmental aspect to be lacking. This might show that the focus on continuous competency development is limited and CBE might not be effectively integrated at the workplace. Summative assessment of individual competencies (step 5) was well established but summative assessment of global professional competence (step 6) was seen as the grading part of work-integrated learning (grade between 1 and 20/20) and not as an opportunity to self-direct the learning process as was intended with the model. Nevertheless, all participants’ groups stated that the third step (self-monitoring performance) and the fifth step (summative assessment of individual competencies) were clearly linked to the predefined competencies.

### Barriers to implement CBE

**Fig. 2:**
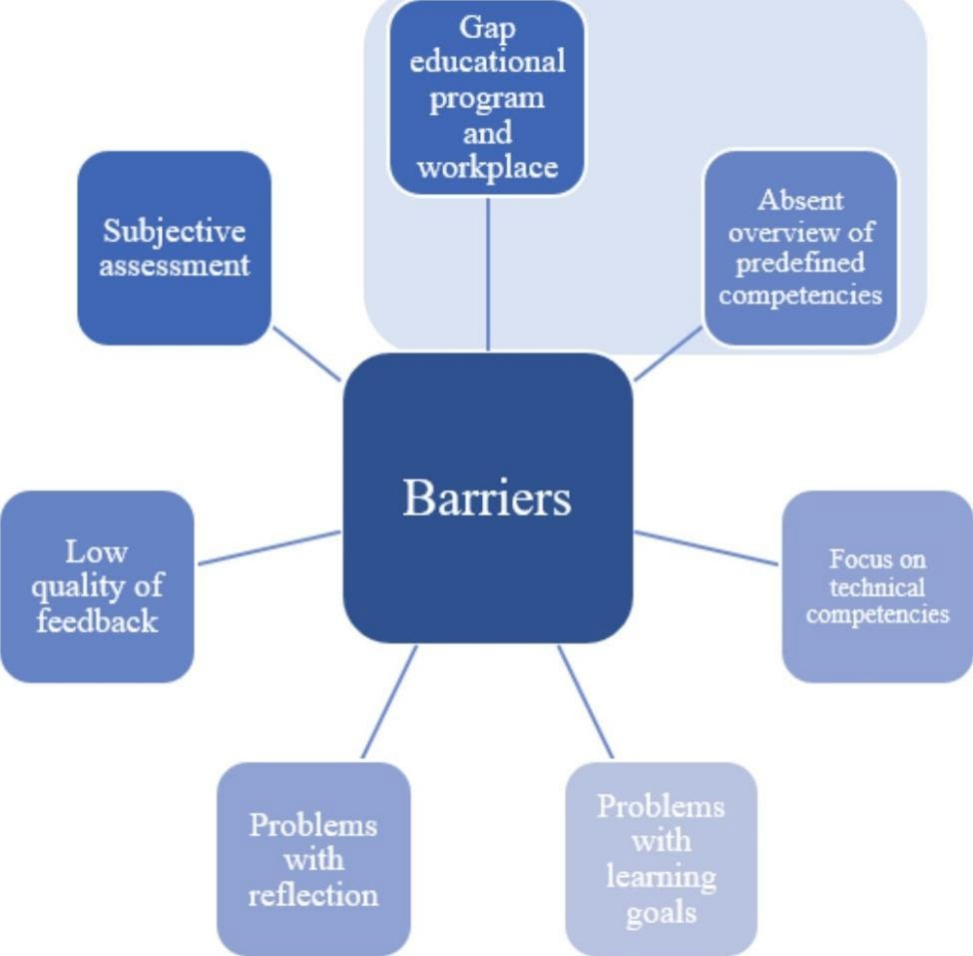
Visualization of the barriers affecting CBE implementation

Further analysis of the results helped identifying seven barriers that might hinder the effective implementation of CBE (see Fig. 2). The following discussion is based on these seven barriers as the identification of potential barriers might offer solutions to remove these barriers and support CBE implementation.

## Discussion

This study explored how students, mentors, and educators from six different healthcare disciplines in Flanders (Belgium) perceived the implementation of CBE in current practice at the workplace. We conducted semi-structured focus group interviews using the six-step model of Embo et al. (2015). The model was adopted to structure the interviews and to study CBE building blocks. The stakeholders in this study perceived that CBE was unsuccessfully implemented hindering the continuity of work-integrated learning and resulting in fragmentation of the learning process. The analysis resulted in the identification of several barriers to its implementation at healthcare workplaces (see Fig. 2).

**Gap between the educational program and the workplace***(step 1: competency selection)*.

Students were hardly aware of the nature of CBE. Also, mentors at the workplace didn’t feel informed about the role of competencies within the curriculum, and their roles in fostering this competency development. Due to the lack of linkage between predefined competencies and the practice experiences, students did not understand very well why they had to start from predefined competencies (step 1). This is in contrast with the aim of CBE to support the learning process by starting from operational outcomes [9, 46, 47]. This challenge was already acknowledged by Bharj & Embo (2018). Moreover, the premise of CBE is that students transfer their focus upon competencies developed within their educational program to the mastery of these same competencies within the workplace. But as reflected in the study results, competencies are hardly transferred automatically [49]. Parmar et al. (2021) also identified this problem and formulated it as ‘ a lack of information about the innovation’ to explain why it is a barrier for successful implementation [50]. Thomas et al. (2017) also described this problem and stated that educators saw that the implementation of evidence-based approaches might not have the desired impact on education and healthcare [51].

Overall, the above findings confirm that CBE-implementation is difficult. When we look at Roger’s DOI research, it might be good to provide transparent information about the nature of the CBE curriculum, and for mentors and educators to be jointly involved in the educational trajectory might improve the link between a CBE curriculum and the internship experience. The importance of communication and the social environment in explaining the theory-practice gap was also reflected in the perspectives of stakeholder groups. Educators in our study were well-informed about the predefined competencies whereas mentors were hardly aware of the competency framework at play. Educators emphasized the priority of predefined competencies while students and mentors reported they experienced an exaggerated focus on these competencies, especially the technical ones. These discrepancies help partly clarifying the gap between theory (educational program) and practice (workplace). Although CBE seems an established practice, our findings suggest that insufficient attention is being paid to the main elements described above. Parmar et al. (2021) identified possible solutions: (1) creating an intentional implementation plan whereby the plan has to be adapted to each specific context (e.g., adapting the competency framework to each of the educational programs and create a plan to disseminate this framework to healthcare workplaces); (2) optimizing the sharing of information with the workplace (e.g., by providing information exchange meetings at healthcare workplaces to inform mentors); (3) providing organizational support where a lack of time can be solved by the provision of sufficient staff and/or more effective information technology; and (4) an interactive training program which facilitates offering tailor-made training programs to each stakeholder [50].

**Absent overview of predefined competencies***(step 1: competency selection)*.

The second barrier is that the workplace is often not aware of the educational requirements. Students perceived that an overview of the predefined competencies was hard to find. Moreover, some mentors were often not aware that an overview even existed. The difference with the first barrier lies in the fact that the first barrier is related to the different roles within education programs, and curriculum design and transfer while the second has to do with practical issues as a competency overview should be easy to find; e.g., in a student’s ePortfolio. In principle, a competency overview is a cornerstone for stakeholder involvement. How can CBE be implemented in practice if students and mentors are not completely aware of the predefined competencies? It might affect the work-integrated learning process since it easily leads to a limited focus on competency development. For instance, students indicated that monitoring competency development was marred by shortcomings in work-integrated learning assessment instruments as they are often difficult to use and not specific enough. Effective assessment instruments require a clear overview of the predefined competencies [48, 52].

**Focus on technical competencies***(step 1: competency selection; step 2: formulating learning goals; step 3: self-monitoring performance; step 4)*.

The focus on technical competencies might be problematic. As van der Vleuten (2015) stated: generic competencies are essential for CBE implementation and the focus on generic competencies might improve the quality of patient services and consequently healthcare [7]. The focus on technical competencies when formulating learning goals but also during reflection, feedback, and assessment affects the potential to guarantee a focus on continuous competency development [29]. Our findings confirm what authors identified in ePortfolio research: there is a dominant focus on achieving technical competencies rather than on a comprehensive assessment of all competencies [53, 54]. An explanation for this narrow technical focus could be that giving feedback on techniques is easier. Also, feedback given on generic competencies might be linked to students’ personal characteristics; e.g., taking initiative to collaborate is more difficult for a timid person. Although the overwhelming focus on technical competencies, the importance of generic competencies is gaining interest. That is made clear by the cross-disciplinary use of the concept ‘T-shaped professional’ described by Gardner & Estry (2017). The concept ‘T-shaped’ was first introduced by David Guest in the early 1990s where it was used to describe the technology-savvy employee that would be needed in the immediate future [45]. In the current study, all stakeholders perceived the importance of the generic competencies as at least as important as the more specific, technical competencies. This finding highlights the importance of training ‘T-shaped professionals’ emphasizing a holistic and practice-based vision on CBE. As CBE currently fails to increase the focus on generic competencies in practice, the implementation of Entrustable Professional Activities (EPA) might offer opportunities. These EPAs are no alternatives to replace competencies but they facilitate the translation of competencies into practice by integrating them into ‘professional activities’ [55]. To illustrate, if a healthcare professional can perfectly execute a patient handover, he has to master several competencies. In the context of the ACGME competency framework, he has to master four competency domains: Medical Knowledge, Patient Care, Interpersonal skills and Communication, and System-Based Practice [55]. Thus, EPAs require the integration of multiple competencies aligning with what healthcare professionals do in practice and could be complementarily used with the competencies that are already used within the educational program [56, 57].

**Problematic formulation of learning goals***(step 2: formulating learning goals)*.

The finding that the formulation of learning goals is problematic, is in accordance with a study of Cho et al. [58]. One of the problems that recurred in this study was that students engaged in a rather artificial way with learning goal formulation because it was mandatory. They wrote learning goals without thinking deeply about the nature and requirements of predefined competencies. This weak link between learning goals and predefined competencies might lead them to miss out on learning opportunities. This problem might even be made worse by: (1) the fact that educators and mentors might not be aware of the competencies that students want to achieve during a specific internship and (2) the fact that they lack time to guide students during the learning goal formulation. Cho et al. (2017) emphasized how (novice) students needed time and guidance during this process.

**Problematic reflection activity***(step 4: self-monitoring performance; step 5: self-assessing competency development)*.

In relation to step 4 and 5, two problems were identified in our study: (1) no reflection on competency *development*, and (2) no *deep* reflection.

First, the results revealed that reflection on competency development was lacking. This confirmed previous research of Embo et al. (2014) who compared two types of reflective writing activities and observed to a larger extent reflection-on-action as compared to reflection-on-progress. This can be explained since reflection-on-action is directly related to day-to-day improvements and feels very operational. Although reflection-on-progress is important, it is more abstract and difficult to observe and tackle in daily practice [29, 59, 60]. Future research about the role of reflection on competency development to support work-integrated learning might offer opportunities to improve CBE implementation.

Secondly, it seemed that students seldomly reflected deeply. Four possible partial explanations can be put forward:

1. Students stated being afraid of reflecting too open and deeply because assessment activities might build on these reflections. This reiterates the findings of Bok et al. (2013) who noted that formative assessment input should not be seen as feedback or assessment *for* learning, but rather as assessment *of* learning [52]. Reflection needs to be stimulated without students being judged by using these reflections for assessment. As already stated three decades ago, visions might be need to change from assessment *of* learning to assessment *for* learning [61, 62].
2. According to students, there was a mandatory educational requirement to reflect upon each individual competency. In case a reflection was missing for a certain competency, students could not be assessed. As a consequence, reflections were written for each competency even if no competency-relevant experiences were present. Previous research confirmed this finding where students write down reflections because it is required or write ‘tick-box’ reflections lacking deep reflection processes [63].
3. The developmental angle that could link reflections based on a series of learning experiences within one internship or within a series of internships lacks in practice according to participants. Nevertheless, this developmental aspect might support a holistic and deeper reflection.
4. Previous studies emphasized that students focus on patient care over learning which might hinder deep reflection on these patient care experiences and effective work-integrated learning [64].

**Low feedback quality***(step 4: self-monitoring performance; step 5: self-assessing competency development)*.

Feedback was emphasized as being a key aspect of the learning process. This is in line with the core of the CBE approach [52, 65]. Unfortunately, several problems were perceived related to giving or receiving feedback that resulted in low feedback quality. The largest barrier was the lack of time to provide feedback. Students in our study perceived limited guidance due to the lack of time available to educators and mentors. Oudkerk Pool et al. [66] confirmed that a lack of time often causes a lack of guidance. The lack of time might be caused by the high workload that was experiences at the workplace [50]. This lack of time might be seen as a shared problem of different stakeholders in the work-integrated learning setting [65] and needs to be tackled so that educators and mentors are more engaged in feedback interactions [67]. This is critical since a lack of time contradicts the nature of reflection and guidance as conditions for learning at the workplace (Embo et al., 2015). Moreover, educators and mentors stated during the interviews that the fear to demotivate or hurt students’ feelings hindered them to give complete qualitative – including negative - feedback. Giving feedforward instead of feedback might offer opportunities by giving options to improve future performances [68]. But, the lack of expertise of mentors in giving feedback and/or feedforward might hinder this process. The emphasis on mentor training fits the agenda of earlier research [52]. Lastly, the low feedback quality could be explained by the lack of an overview of predefined competencies [67]. Effective feedback encompasses current and future behavior and addresses the broader picture, which leads us back to the need to emphasize holistic competency development.

**Subjective assessment***(step 6: global professional competence)*.

A possible reason for the existence of subjective assessment could be that there were no clear behavioral indicators to assess the predefined competencies [65]. There was also found a weak correlation between students’ grade and educators’ judgements in previous research [65]. This subjectivity risk can be tackled by building on rubrics for each competency. This presents assessors with descriptive information to position observed behavior along a broader competency development scale [69]. Studies also stated that a mastery baseline should be provided to guide judgment. Otherwise, stakeholders are not aware of the mastery level expected at a certain moment in an internship.

Next to the seven barriers complicating CBE implementation, a strong link between most steps in the six-step model was missing and the predefined competencies were not used throughout the learning process. Nowadays, ePortfolios might offer opportunities to capture competency development and support continuity, not only within internships but also between internships and after graduation [70]. Future research might build on this finding by exploring the opportunities of ePortfolios in supporting competency development. The missing step four where reflection on competency development is central confirms that the developmental aspect is still lacking in work-integrated learning although this might be seen as a cornerstone of CBE. However, the fact that after all these years, an effective implementation seems to lack, might raise the following question: “Is it the eligibility of a CBE approach that does not fit the work-integrated learning processes at healthcare workplaces or is it the fact that the CBE approach is still not effectively implemented at healthcare workplaces?”. Or perhaps the statement of Van der Vleuten in 2015 still applies in 2022: “if we truly embrace competency-based education, there is still a long way to go” [7] because nowadays ‘theory beats practice’ as CBE has not yet been effectively implemented.

### Limitations

The generalizability of our findings might have been affected by the size and nature of the sample: fifteen participants from different programs set up in the Flemish context. Nevertheless, the involvement of three different stakeholder groups and different healthcare disciplines helped collecting rich and multidisciplinary data from work-integrated learning settings in Flanders (Belgium). Future research might start from our findings and involve other stakeholders, from other professions and from other contexts or settings. Furthermore, we did not do a member check so a confirmatory round was absent. Substantively, the CBE implementation was only explored during work-integrated learning at healthcare workplaces and not during the full curriculum because this was not the scope of our study. Moreover, this study did not zoom in on possible guidance models that educators might opt in practice to overcome the gap between theory and practice. Moreover, this study focused especially on actual CBE implementation and related barriers while the focus on CBE implementation also offers opportunities to study topics about e.g., quality of care, patient involvement, etc. in future research. At last, this study did not zoom in on possible practice-related guidance models that educators might adopt to overcome the gap between theory and practice.

### Future research

Further research is needed to further investigate CBE implementation issues at healthcare workplaces. Our findings and discussion emphasize the need for a predefined overview of competencies, an emphasis on engaging all stakeholders in the initial CBE implementation, and the development of work-integrated learning assessment instruments with concrete and competency-based behavioral indicators. Moreover, the opportunities of ePortfolios to capture competency development during the full educational program as well as after graduation constitutes an interesting research track. Last, future studies could center on ways to address and assess generic competency development during work-integrated learning, such as communication competencies.

## Conclusion

Healthcare educational programs often build on a CBE approach. The present study explored the perceptions of students, mentors, and educators about such CBE implementation in the workplace during work-integrated learning. Our findings suggest that current practices fail in terms of actual CBE implementation. CBE implementation starts and ends with an overview and selection of relevant competencies. But in the Flemish setting, the starting focus seems lacking which causes problems with implementing the subsequent building blocks of CBE; e.g., self-assessing competency development and the linkage between the steps of the model of Embo et al. (2015). This could only be remediated by – among others - effective sharing of information, a thorough implementation plan, and interactive training methods. Further research is necessary to investigate how CBE implementation can be improved to achieve optimal work-integrated learning in healthcare workplaces so that theory can ‘meet’ practice instead of ‘beat’ practice when it comes to CBE implementation.

## Electronic supplementary material

Below is the link to the electronic supplementary material.

Supplementary Material 1

## Data Availability

A Data Management Plan was constructed via https://dmponline.be and monitors the storage and access to the data (ID: 107,491). Data are available on request by contacting the main author (Oona.Janssens@UGent.be).

## List of Abbreviations

CBE: Competency-based education
CanMEDS: Canadian Medical Education Directives for Specialists
ACGME: Accreditation Council for Graduate Medical Education
EPA: Entrustable Professional Activity
