# Supplementary material for: When theory beats practice: the implementation of competency-based education at healthcare workplaces: Focus group interviews with students, mentors, and educators of six healthcare disciplines
Source: BMC Med Educ. 2023 Jun 29;23:484. doi: 10.1186/s12909-023-04446-3 (PMC10308607; doi:10.1186/s12909-023-04446-3)
Supplement: Supplementary file 1 — Supplementary Material 1 [file 12909_2023_4446_MOESM1_ESM.docx]

**Supplementary material**

***Appendix 1: Codebook focus group interviews with mentors***

Main themes, sub-themes (level 1), sub-themes (level 2), sub-themes (level 3) and codes are shown in the Table below.

| ***Main themes – Sub-themes level 1 – Sub-themes level 2 – Sub-themes level 3 – Codes*** |
| --- |
| Administrative Organization of Internships |
| Organization of hours of internships |
| Communication between student-institution-workplace |
| Contact with educator |
| Contact mentor and educator |
| Contact workplace - institution |
| Bad communication between student and mentor/educator |
| No transparant communication between student and mentor/educator |
| New system of work-integrated learning more from the workplace than from the education program |
| Personal coach from the educational program |
| Internship office – internship guidance – confusion |
| Internship guide |
| Templates for Assignments |
| Template strong and weak characteristics |
| Template reports |
| Work-integrated learning is seen as different to internships |
| Work-integrated learning as a specific part of the educational program |
| Characteristics of Good or Bad Mentors |
| Opinion of student about mentor |
| Mentor as coach or assessor |
| Mentor as educator |
| Disadvantages of mentors being educators too |
| Mentors are motivated nurses |
| Organization mentor training |
| Content of mentor training |
| Problems with mentors |
| Mentors have to few skills to give good feedback |
| Difficulties of having more than one mentor |
| Difference between being a mentor and being no mentor |
| Communication Competency |
| Attitude and communication competency are innate |
| Communication competency: content |
| Communication competency: growth |
| Communication competency: growth after graduation |
| Guidance of Students during Internships |
| One on one work with student |
| Conversations between student and mentor |
| Way of guiding students in difficult situations |
| Stress moment of students |
| Time investment of mentors |
| Burden of mentors because of students |
| Pressure of being a mentor |
| Lack of time of mentors |
| Time investment of students |
| Time investment difference between paper portfolio and ePortfolio |
| Time investment of educator |
| Lack of time of educators |
| Mentor’s high expectation of students |
| Preparation strong and weak points at the start of an internship |
| Working with the student at the workplace |
| Disadvantages |
| Advantages |
| Impact of the place of the internship |
| Types of workplaces |
| Experience of student depends on mentor |
| Differences between workplaces |
| Lifelong learning |
| Competency growth is important after graduation |
| Lifelong learning due to being a mentor |
| Portfolio use by employers |
| Portfolio use after graduation |
| Newly graduated nurse |
| Continuous professional development newly graduated healthcare professional |
| Assessment after three months of work |
| Feedback portfolio newly graduated healthcare professional |
| Feedback portfolio newly graduated healthcare professional (2) |
| Amount of use of portfolios by newly graduated healthcare professionals |
| Content of portfolio of newly graduated healthcare professional |
| Time of portfolio completion by newly graduated healthcare professional |
| Step 1 - Competency Selection |
| CanMEDS competency framework |
| CanMEDS competency framework as a more usable framework |
| Competencies |
| Importance of predefined competencies for students from the perspective of mentors |
| Describing predefined competencies at the workplace |
| Competency assessment |
| Generic competencies are equal to technical competencies |
| Competence of educators |
| Concept of competency: competencies are seen as ‘talents’ or ‘gifts’ |
| Competency growth |
| Communication growth |
| Possible competency growth by formulating learning goals |
| Competency growth of students important for potential employers |
| Competency growth newly graduated healthcare professional |
| Lacking continuity: Competencies are not taken to next internships |
| Evaluating competency growth |
| Evolution of students during an internship |
| Overview assessment of competencies of student – mentor – educator |
| Displaying expectations of competency growth at the start of an internship |
| Who can see which competencies are attained or not at following internships? |
| Competency profile associate degree nursing |
| Difference between the importance of competencies or treatment plan |
| Difference between competencies of bachelor nursing and associate degree nursing |
| Predefined competencies in learning goals |
| Predefined competencies only at time of assessment |
| Asking feedback as a competency |
| Too little emphasis on personal competencies (not from the educational program) |
| Selection of competencies by students |
| Competencies to be attained |
| Focus on technical competencies |
| Hard to find overview of predefined competencies |
| (Too much) weight of specific competencies |
| Too little focus on predefined competencies |
| Use of Entrustable Professional Activities |
| Strong and weak points are seen as competencies |
| Step 2 - Formulating Learning Goals |
| Learning goals |
| Adaptation of learning goals to workplace |
| Action plans as learning goals |
| (Too little) feedback on learning goals by mentors |
| (Too little) feedback on learning goals as educators according to mentors |
| Discussing learning goals with mentors |
| Training the formulation of learning goals from the education program |
| Learning goals are confused with competencies |
| (Not) taking (enough) learning goals to next internships |
| Assessment of the formulation of learning goals |
| Step 3 - Self-Monitoring Performance |
| Evaluating reflections and the way of reflecting |
| Daily reflections are not seen as necessary |
| Learning moments |
| Learning to reflect during the educational program |
| Reflection and feedback at home |
| Superficial reflection by students |
| Problems due to low quality of reflections |
| Reflection: content |
| Reflection on paper |
| Linking reflections on specific learning moments to the predefined competencies |
| Reflection systems and rules |
| Self-reflection guidelines |
| Guidelines are not necessary as a student |
| Reflection at home as a student |
| Reflection methods |
| Reflection report |
| Case study report |
| Less reflection reports in future |
| SWOT analysis as reflection report |
| Reports on the basis of Entrustable Professional Activities |
| Mentors stimulate reflection by students |
| Too little and too little deep reflection |
| Self-reflection |
| Amount of times of self-reflection |
| Step 4 - Self-Assessing Competency Development |
| Barriers of giving good feedback |
| Lack of time for giving feedback |
| Guidance from the educational program |
| Two guiding moments and only e-mails are too little guidance |
| Extra contact between school and workplace in case of problems |
| Matching student with workplace |
| Transition to process guidance instead of workplace guidance |
| Educator has no insight into what happens at the workplace |
| Student sends information e-mail to educator |
| Process coach from the education program |
| Reflection reports only for educational program – not obligated |
| Change in guidance from the educational program |
| Keeping feedback forms |
| Feedback |
| Deep feedback in mental healthcare |
| Reading feedback of educators as a mentor |
| Giving feedback as a mentor |
| Mailing feedback to students |
| Giving (quick) oral feedback to students at the workplace |
| Feedback on attitude, understanding and communication |
| (Too much) feedback on techniques |
| Feedback on reflection |
| Feedback oral or written |
| Writing feedback as a mentor |
| Feedback seen as formative or summative |
| Giving feedback as an educator according to mentors |
| Feedback about the guidance of mentors |
| Feedback in a portfolio of newly graduated healthcare professionals |
| Reading feedback of mentors by educators |
| Asking feedback as a student |
| Moments when feedback can be requested as a student |
| Difficulties with receiving feedback of mentors as a student |
| The use of feedback of mentors for the assessment |
| The use of feedback of educators for the assessment |
| Lack of time to give feedback |
| Feedback on content is given by mentors |
| Mentors ask students to first write feedback down |
| Mentors don’t want to hurt a student’s feelings |
| Mentors want to motivate students |
| Writing oral feedback is not always easy |
| Not receiving and writing down feedback causes difficult assessments |
| Superficial feedback by mentors |
| Quickly writing down feedback |
| Quickly writing down feedback by students |
| Too little feedback of educators according to students |
| Writing feedback at home |
| Difference between feedback on paper and digital |
| Careful feedback of mentors |
| Growth in reflections |
| Growth portfolio |
| Growth portfolio by students |
| Growth portfolio is being forgotten |
| Mentors have too little skills to give good quality feedback |
| Personal development |
| Not recognizing the emotions of students during by the educational institution |
| Professional development of the student as a course |
| Skilled companionship – personal growth during internships |
| Only time for reflection during assessments |
| (Too little) reflection on competency growth |
| Training of mentors to give good quality feedback |
| Internal training of mentors at the workplace |
| Content of the training |
| Timing of the training |
| Amount of training |
| Self-assessment |
| Step 5 - Summative Assessment of Individual Competencies |
| Assessment |
| Share of mentor during assessment |
| Global assessment of internship by different assessment moments |
| (too little) clear assessment criteria |
| Final decision usually goes well |
| Final assessment |
| Assessment using a scale |
| Assessment by mentor, not executive healthcare professional |
| Assessment in associate degree nursing programs |
| Share of all parties during assessments |
| Assessment after three months of work |
| Assessment of competency growth |
| Assessment of portfolio itself |
| Use of feedback of mentor for assessment |
| Use of feedback of educator for assessment |
| Mentors have no share in giving grades |
| Right grade is important |
| Skill list is used |
| Not writing down feedback causes difficulties during assessment |
| Unclarity about who gives grades |
| Discrepancies between the grades of mentors and educators |
| Giving grades is not necessary for students according to mentors |
| Giving grades |
| Giving grades as a mentor |
| Internship reports are not reflection reports |
| Summative assessment of predefined competencies |
| Mid-term assessment |
| Digital mid-term assessment |
| Responsibility of giving grades of mentors and educators |
| Different assessment for technical and generic competencies |
| Who completes the assessment forms? |
| Variety in systems, documentation, feedback and assessment |
| No uniform assessment criteria |
| No uniform documents |
| No use of an ePortfolio |
| No uniform system |
| No uniformity between educational programs and institutions |
| Standardized form for reflection and feedback |
| Necessary uniformity for forms |
| No uniform digital media |
| No uniform form for feedback and assessment in the past |
| Changing internship assessment |
| A lot of change in internship assessment |
| Little change in internship assessment |
| Step 6 - Summative Assessment of Global Professional Competence |
| (Too little) competency growth |
| Summative assessment of predefined competencies |
| The use of ePortfolios |
| Wait and see how the ePortfolio works |
| Barriers when using ePortfolios |
| Extra time burden when using ePortfolios |
| Disadvantages feedback and assessment of newly graduated healthcare professionals |
| Paper or PC not present |
| Setting is crucial |
| Using a(n) (e)Portfolio |
| The use of Medbook |
| Implementation of an ePortfolio |
| Disadvantages of using Medbook with a group account |
| Training to use an ePortfolio |
| Different view of ePortfolio by student, mentor, and educator |
| Medbook is used |
| Taking the ePortfolio to next internships |
| Disadvantages |
| Onedrive as an ePortfolio |
| Training to use an ePortfolio |
| Access to the ePortfolio |
| Access of mentor to the ePortfolio of a student |
| Use of internship documents by mentors |
| Access to internship documents by mentors |
| Access to reflection and feedback by mentors |
| Access to reflection and feedback by educators according to mentors |
| Types of (e)Portfolio systems |
| Digital platform |
| Paper portfolio |
| Feedback and reflection on paper |
| Assessment on paper of newly graduated healthcare professionals |
| Portfolio as an internship folder |
| 'Portfolio’ on paper but input (e.g. feedback) digital |
| Feedback portfolio for newly graduated healthcare professionals |
| Writing down feedback now and then |
| Explaining the use of the ePortfolio |
| Expectations towards ePortfolios |
| ePortfolios need to be time efficient |
| ePortfolios might offer solutions |
| Availability of PC is necessary at the workplace |
| Choosing the content of the ePortfolio after graduation |
| Advantages of ePortfolio use |
| Overview of assessment and progress of students |
| Advantages Medbook |
| Advantages of using a rubric |
| The contexts were ePortfolios can be used in |
